# Genome Sequence of a Cluster CR2 Gordonia terrae Phage, StarStruck

**DOI:** 10.1128/mra.00694-22

**Published:** 2022-08-30

**Authors:** Wyatt Cannell, Eleanor Carrolton, Julia Coombs, Addison Gambol, McHenna Martin, Melody Neely, Sally Molloy

**Affiliations:** a Molecular and Biomedical Sciences, University of Maine, Orono, Maine, USA; b The Honors College, University of Maine, Orono, Maine, USA; Queens College CUNY

## Abstract

Bacteriophage StarStruck is a lytic *Siphoviridae* phage that infects Gordonia terrae 3612. The 68,128-bp genome of StarStruck has a GC content of 65.4% and contains 92 protein-coding genes, including the gene for a HicA-like toxin. StarStruck was assigned to subcluster CR2 based on >35% shared gene content with other cluster CR genomes in the Actinobacteriophage Database.

## ANNOUNCEMENT

Actinobacteriophages, which are viruses that infect bacteria of the phylum *Actinobacteria*, are extremely abundant and diverse (1–4). By characterizing lytic bacteriophages, we increase our understanding of phage diversity and susceptibility, which is relevant to applications such as phage therapy and biological control of foaming bacteria in wastewater treatment plants (5, 6). StarStruck was isolated from a soil sample collected at 27°C in Orono, Maine (44.896455N, 68.66614W), using Gordonia terrae 3612 (7). Soil extracts were prepared in peptone-yeast extract-calcium (PYCa) medium and filtered on 0.22-μm filters before inoculation with G. terrae and incubation at 30°C for 2 days. The extract was diluted and plated onto PYCa agar in soft agar containing G. terrae, and plaques were purified by four rounds of plaque assays (7). After incubation for 2 days at 30°C, StarStruck formed 4-mm clear plaques with turbid edges on a lawn of G. terrae, but lysogen isolation techniques did not yield stable lysogens (7). The particle morphology of StarStruck was determined by negative staining transmission electron microscopy. StarStruck has a *Siphoviridae* morphology with a 65-nm (standard error [SE], ±3.2 nm) icosahedral head and a 297.5-nm (SE, ±7.5 nm) flexible, noncontractile tail (*n* = 3 particles).

DNA was extracted from a high-titer lysate by phenol-chloroform extraction and prepared for sequencing using the NEBNext Ultra II library preparation kit (New England BioLabs, Ipswich, MA) (8). Sequencing on an Illumina MiSeq platform yielded 561,846 single-end 150-bp reads. Newbler v2.9 and Consed v29 (9) were used to assemble raw reads and check for completeness, yielding a 68,128-bp genome with a GC content of 65.4%. Genome ends are defined by single-stranded 10-bp 3′ extensions (CGCCGCGTAC). StarStruck shares >35% gene content with members of cluster CR in the Phamerator database Actino_Draft and was assigned to subcluster CR2 (4, 10, 11).

**FIG 1:**
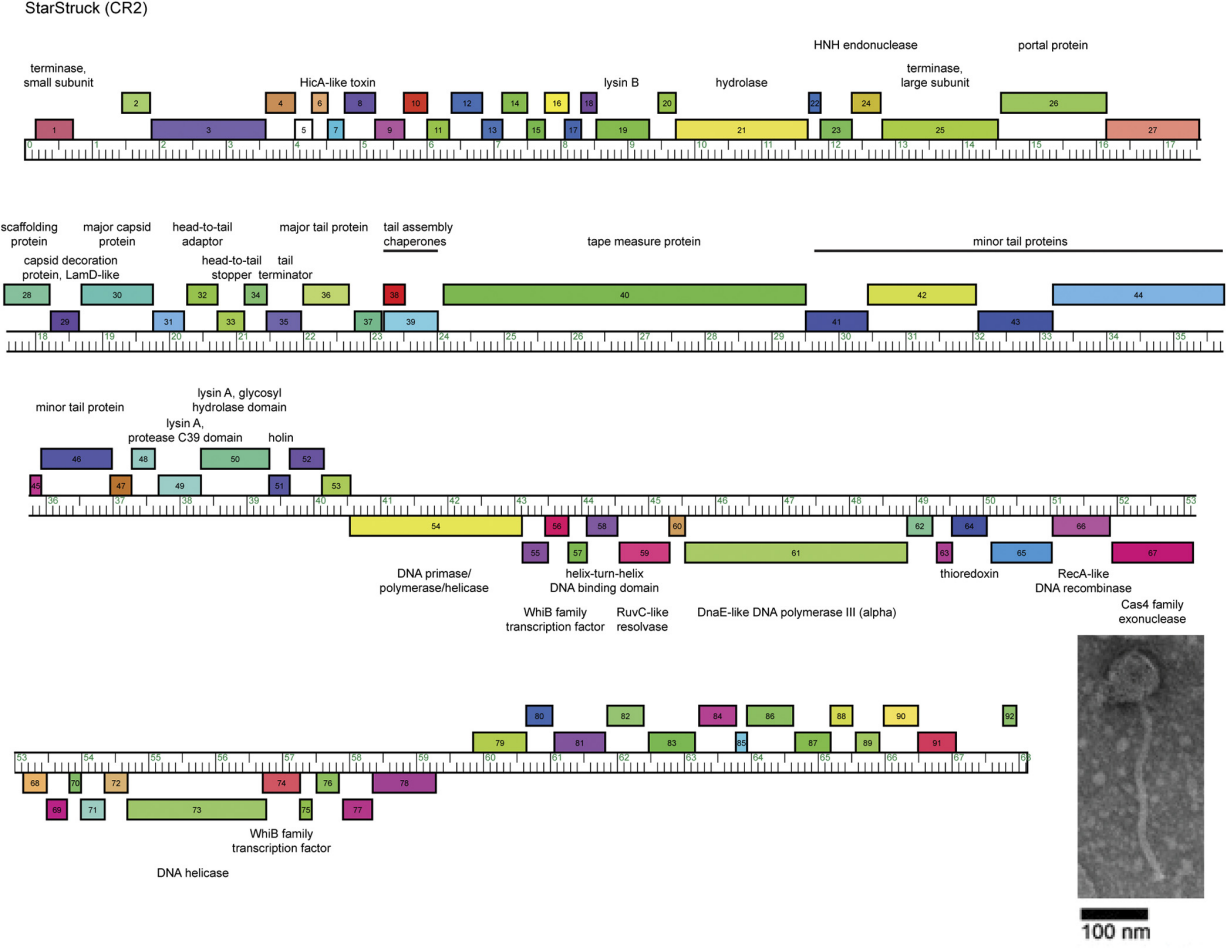
Genome map of *Gordonia* phage StarStruck. The ruler indicates genome coordinates in units of kilobase pairs. Forward and reverse genes are represented by colored boxes above and below the ruler, respectively. Genes were assigned to a phamily using Phamerator (10) with the Actino_Draft database, and different phamilies are indicated by different colors. An electron micrograph of StarStruck is shown in the inset, with a scale bar of 100 nm. The average diameter and length of the particle head and tail were determined by measuring the dimensions of three different particles.

The genome of StarStruck was autoannotated using GLIMMER v3.02 and GeneMark v2.5 within DNA Master v5.23.6 (http://cobamide2.bio.pitt.edu) and PECAAN (https://blog.kbrinsgd.org/) (12, 13). Translational starts were predicted manually based on inclusion of coding potential in GeneMark.hmm and conservation across homologs in BLAST and Starterator (http://phages.wustl.edu/starterator) analyses (14). Putative gene functions were predicted using BLAST, TMHMM, and HHpred, and gene maps were prepared using the Phamerator database Actino_Draft (10, 15, 16). No tRNA genes were identified by ARAGORN v1.2.38 and tRNAscan-SE (17, 18). StarStruck contains 92 protein-coding genes. The left arm of the genome contains forward-transcribed genes (gp1 to gp53) beginning with the small-subunit terminase (gp1) (Fig. 1). The large-subunit terminase and the rest of the structural and assembly genes (gp25 to gp46) continue after an ~12,000-bp region containing 23 genes. The right arm contains reverse-transcribed genes (gp54 to gp92), including two WhiB family transcription factors (gp56 and gp74), a DnaE-like DNA polymerase (gp61), and a DNA helicase (gp73). StarStruck lacks lysogenic genes such as an integrase and an immunity repressor, indicating that StarStruck is a lytic phage (19).

Like many cluster CR phages, StarStruck encodes a HicA-like toxin (gp7) within the 12,000-bp region separating the small- and large-subunit terminases. Another interesting feature of StarStruck is the location of the lysin B (gp19) within this region, rather than adjacent to the lysin A genes (protease C39 domain [gp49] and glycosyl hydrolase domain [gp50]), which are located downstream of the minor tail proteins.

### Data availability.

StarStruck is available at GenBank with the accession number ON456333 and the Sequence Read jhu7 (SRA) accession number SRX14816101.
